# Bacterial Active Community Cycling in Response to Solar Radiation and Their Influence on Nutrient Changes in a High-Altitude Wetland

**DOI:** 10.3389/fmicb.2016.01823

**Published:** 2016-11-17

**Authors:** Verónica Molina, Klaudia Hernández, Cristina Dorador, Yoanna Eissler, Martha Hengst, Vilma Pérez, Chris Harrod

**Affiliations:** ^1^Programa de Biodiversidad and Departamento de Biología, Facultad de Ciencias Naturales y Exactas, Observatorio de Ecología Microbiana, Universidad de Playa AnchaValparaíso, Chile; ^2^Centro de Investigación Marina Quintay, Facultad de Ecología y Recursos Naturales, Universidad Andres BelloValparaíso, Chile; ^3^Laboratorio de Complejidad Microbiana y Ecología Funcional and Departamento de Biotecnología, Facultad de Ciencias del Mar y Recursos Biológicos, Universidad de Antofagasta, AntofagastaChile; ^4^Centre for Biotechnology and BioengineeringSantiago, Chile; ^5^Centro de Investigación y Gestión de Recursos Naturales, Instituto de Química y Bioquímica, Facultad de Ciencias, Universidad de ValparaísoValparaíso, Chile; ^6^Departamento de Ciencias Farmacéuticas, Facultad de Ciencias, Universidad Católica del NorteAntofagasta, Chile; ^7^Fish and Stable Isotope Ecology Laboratory, Instituto de Ciencias Naturales Alexander von Humboldt, Facultad de Ciencias del Mar y Recursos Biológicos, Universidad de AntofagastaAntofagasta, Chile

**Keywords:** extreme ecosystem, 16S RNA gene pyrosequencing, solar radiation, PAR, UVR, phototrophic bacteria, rare microbial biosphere

## Abstract

Microbial communities inhabiting high-altitude spring ecosystems are subjected to extreme changes in solar irradiance and temperature throughout the diel cycle. Here, using 16S rRNA gene tag pyrosequencing (cDNA) we determined the composition of actively transcribing bacteria from spring waters experimentally exposed through the day (morning, noon, and afternoon) to variable levels of solar radiation and light quality, and evaluated their influence on nutrient recycling. Solar irradiance, temperature, and changes in nutrient dynamics were associated with changes in the active bacterial community structure, predominantly by Cyanobacteria, Verrucomicrobia, Proteobacteria, and 35 other Phyla, including the recently described Candidate Phyla Radiation (e.g., Parcubacteria, Gracilibacteria, OP3, TM6, SR1). Diversity increased at noon, when the highest irradiances were measured (3.3–3.9 H′, 1125 W m^-2^) compared to morning and afternoon (0.6–2.8 H′). This shift was associated with a decrease in the contribution to pyrolibraries by Cyanobacteria and an increase of Proteobacteria and other initially low frequently and rare bacteria phyla (< 0.5%) in the pyrolibraries. A potential increase in the activity of Cyanobacteria and other phototrophic groups, e.g., Rhodobacterales, was observed and associated with UVR, suggesting the presence of photo-activated repair mechanisms to resist high levels of solar radiation. In addition, the percentage contribution of cyanobacterial sequences in the afternoon was similar to those recorded in the morning. The shifts in the contribution by Cyanobacteria also influenced the rate of change in nitrate, nitrite, and phosphate, highlighted by a high level of nitrate accumulation during hours of high radiation and temperature associated with nitrifying bacteria activity. We did not detect ammonia or nitrite oxidizing bacteria *in situ*, but both functional groups (*Nitrosomona* and *Nitrospira*) appeared mainly in pyrolibraries generated from dark incubations. In total, our results reveal that both the structure and the diversity of the active bacteria community was extremely dynamic through the day, and showed marked shifts in composition that influenced nutrient recycling, highlighting how abiotic variation affects potential ecosystem functioning.

## Introduction

High-altitude ecosystems are often extreme. The microbial community found at the Salar del Huasco, a high-altitude (>3,800 m asl) wetland located in the Chilean Altiplano, is exposed to a range of extreme conditions. This includes total solar irradiance reaching levels of up to 1,000 W m^-2^ (Aceituno, 1997; de la Fuente, 2014), the effect of which are intensified in these ecosystem due to low atmospheric concentrations of ozone and water vapor, and high ground reflectivity (albedo), but which can be countered by aerosol dust, in this arid desert ecosystem (Cordero et al., 2013, 2016). Salar de Huasco is subject to extreme shifts in temperature through the day (can range from -15 to 20°C) and high evaporation rates, which result in a negative water balance (e.g., precipitation rates of 50–300 mm y^-1^ versus evaporation rates of 600–1200 mm y^-1^ (Risacher et al., 2003; de a Fuente and Niño, 2010; de la Fuente, 2014). The Salar de Huasco is largely groundwater fed, and upwells via a limited number of springs, which generate a wetland habitat, characterized by complex pond and pool systems surrounded by vascular plants forming peatland, locally referred to as bofedales” with the water flowing into a permanent shallow lake (de a Fuente and Niño, 2010).

Aquatic life in this and other similar high-altitude ecosystems in the Andes is adapted to extreme conditions, and also faces temporal changes in salinity and nutrient availability such as nitrogen across seasonal and inter-annual scales (Marquez-Garcia et al., 2009). Microbial life in these ecosystems is notably highly diverse, characterized by novel groups of prokaryotes, and includes the conspicuous presence of microbial mats (Dorador et al., 2008a,b, 2009, 2013). Highland aquatic ecosystems such as the Altiplano and the high lakes of the Tibetan plateau represent hotspots of microbial life, and can be considered as model ecosystems to study the response of microorganisms to environmental gradients (Jiang et al., 2009; Hu et al., 2010; Kang et al., 2010; Albarracin et al., 2015).

Photoautotrophic groups are particularly diverse in high-altitude ecosystems, including cyanobacteria from the orders Oscillatoriales, Nostocales, Pleurocapsales, and Chroococcales found both in the water column and the underlying and sediment associated with tributaries and spring sites from the Salar de Huasco (Dorador et al., 2008b). Furthermore, earlier work has shown the existence of a high phototrophic potential different than oxygenic photosynthetic bacteria at this site using clone survey studies (Dorador et al., 2013). This work highlighted the importance of aerobic anoxygenic phototrophic bacteria, including Rhodobacterales and Sphingomonadales from Alpha-proteobacteria, and other potentially relevant bacteria such as Bacteroidetes and Gamma-proteobacteria in the Salar de Huasco. These classes can also include light-harvesting bacteria that use photorhodopsins as an important mechanisms for heterotrophy in this nutrient-limited environment, as seen in some marine habitats (Fuhrman et al., 2008).

Phototrophic bacteria could potentially be negatively influenced by the high levels of solar radiation encountered at such high-altitude ecosystems. For example, inhibitory effects associated with high incident sunlight, such as UVR damage and migratory effects were observed in stromatolites containing oxygenic photosynthetic bacteria (Cyanobacteria) in the high-altitude Lake Socompa in Argentina (Farias et al., 2013). The activity of heterotrophic microorganisms (secondary productivity) was shown to be subject to higher sunlight inhibition at spring sites compared to open water sites (ponds and lakes) with longer solar radiation exposure (for Salar del Huasco, Hernández et al this issue). In other (mainly marine) ecosystems, microbial composition, photohistory, availability of organic matter, susceptibility of competitors to sunlight, bacterivores, and phages have all been identified as significant factors affecting microbial response to solar radiation (Ruiz-Gonzalez et al., 2013). Here, using 16S rRNA (pyrolibraries – cDNA), we explored how the active bacterial community responded to different levels of solar irradiance through the diel cycle (morning, noon, afternoon) and furthermore, using an experimental approach, we showed how the community responded to differential solar spectra and the subsequent effects on nutrient dynamics.

## Materials and Methods

Salar de Huasco (20° 18′S, 68° 50′W) was visited during August 2011 (Austral winter; **Supplementary Figure S1**) as part of the field campaign MOSE 1 “Microbial Observatory of Salar Experiments,” a series of field studies that have run from 2011 until the current time. Sampling and experiments were conducted at a site (H0) located close to an upwelling spring and the start of a stream, which has been sampled for microbial diversity surveys since 2005 (Dorador et al., 2008b).

The response of bacteria to different light spectra was experimentally studied using incubation squared bottles (**Supplementary Figure S1A**), following previous experiments (Hernández et al., 2006, 2007). Water samples from the spring site H0 were distributed in four squared UV transparent plexi-glass (Rhom and Haas) bottles (60 cm × 60 cm × 6 cm height; volume 12 L), for the following treatments: (i) FS (full sun), consisting of an uncovered bottle exposed to natural solar radiation receiving between 280–700 nm according to plexi-glass optical properties, (ii) Dark, bottle covered by aluminum foil and black plastic bags, (iii) PAR (Photosynthetically Active Radiation), bottle covered by Ultraphan UV Opak filter characterized by 50% transmission <395 nm, meaning that they are only PAR transparent (receiving between 400–700 nm), and (iv) PA (PAR and UVA radiation), bottle covered with Montagefolie filter characterized by 50% transmission <320 nm, thus receiving between 320–700 nm. When filters are used the bottle was completely cover. The optical properties of all the materials used here were checked, and have been used in previous studies (Figueroa et al., 1997).

The bottles, one for each treatment, were covered with water and incubated at the same site to maintain similar water temperatures (**Supplementary Figure S1A**). We restricted our experimental design to one site, and to a single (large) bottle per experimental treatment for a mixt of scientific and logistical reasons. The use of a single bottle per treatment in one location permitted us to complete subsampling in a timely manner, an important consideration in this extreme ecosystem. Initial measures of experimental parameters (physical, chemical, and filters to collect microbial community) were taken at 9:30 a.m. and then each bottle was sampled after 2, 6, and 8 h through a diel sun cycle until 17:30 p.m. Conductivity, temperature, and dissolved oxygen concentrations were determined *in situ* and in the incubation bottles before sampling using sensors available from a Thermo Scientific Orion Star Multiparameter (model A329). Incident solar radiation was determined before sunrise (7:10 h) and during each hour after sunrise (7:30–17:30 h) at ground level using a Apogee PAR MQ-200 Quantum Meter. Radiation (W m^-2^) and radiant exposure dose (KJ m^-2^) were also estimated, however, as we did not have access to a data logger, our estimates may be biased.

Sampling consisted of removing ∼1 L of water from each bottle manually, which was transferred to a carboy for subsequent determinations. Water (∼100 mL) for nitrate, nitrite, and phosphate determinations was filtered through 25 mm GF/F filters using a syringe and filter holder, and then stored frozen (-20°C) until analysis (three pseudo-replicates, 15 mL each). Nutrients were analyzed in a Seal segmented flow analytical AutoAnalyzer AA3, following standard colorimetric methods (Laboratorio de Biogeoquímica, Universidad de Concepción, Chile). Bacteria were sampled by filtering water (60 mL, two pseudo-replicates) onto 0.22 μm 25 mm diameter hydrophilic PVDF filters (GVWP02500, Millipore) in the field using a sterilized syringe and filter holder. Filters were preserved with RNAlater^®^ solution (Ambion, Austin, TX, USA) and stored in liquid nitrogen in the field and transferred to -20°C until subsequent RNA extraction in the laboratory. Unfortunately, filters associated with FS (17:30 h) was lost during transport.

### Nutrient Dynamics: Nitrate, Nitrite, and Phosphate Rates of Change through Time

Nutrient dynamics in the different treatments were characterized as rates of change over time (μM h^-1^). These rates were calculated using a comparison of initial compared with nutrient concentrations in the different treatments and times, using an end point approach (Lam et al., 2004; Molina et al., 2005):

$$\mathrm{Nutrient}\;\mathrm{Rate}(\mu Mh^{-1})\;=\frac{\begin{array}{c} {\left[\mathrm{Final}\;\mathrm{Nutrient}\;\mu M\;\mathrm{Treatment}\right]}_{\mathrm{FS},\;\mathrm{PAR},\;\mathrm{PA},\;\mathrm{Dark}}\;\\ -[\mathrm{Initial}\;\mathrm{Nutrient}\;\mu M_{\mathrm{in}\;\mathrm{situ}}] \end{array}}{\mathrm{time}(2h,6h,8h)}$$

Standard error was estimated based on analytical replicates (*n* = 3) within each treatment. Here, a positive value indicates a net accumulation of a given nutrient, whereas a negative value indicates a net consumption of that nutrient.

### RNA Extraction and Molecular Methods

RNA was extracted using Ambion^®^ RNA extraction kit (AM1560) following the manufacturer’s specifications with the addition of a mechanical disruption step, using homogenizing 200-μm-diameter zirconium beads (Low Binding Zirconium Beads, OPS Diagnostics) for two steps of 30 s (∼3,000 rpm) using a Mini-Beadbeater-8^TM^ (Biospec Products). The concentration and quality (A_260_/A_280_ ratio) of RNA extracts was determined spectrophotometrically (Synergy Mx Microplate Reader, BioTek Instruments). DNA traces were removed using the TURBO DNA-free^TM^ kit (Applied Biosystems).

### Analysis of the Structure of the Active Bacterial Community

Bacteria were considered as active based on their detection by high-throughput pyrosequencing method from 16S rRNA gene using cDNA as a template following previous studies, e.g., (Campbell and Kirchman, 2013). cDNA was generated using random primers provided by the ImProm-II^TM^ Reverse Transcription System (Promega Corp.). Bacterial 16S rRNA gene pyrolibraries (V1–V3 region, 491 bp) were generated from these cDNA preparations with the primers 28F (5′-GAGTTTGATCNTGGCTCAG) and 519R (5′-GTNTTACNGCGGCKGCTG) at the Research and Testing Laboratory (RTL, Lubbock, TX, USA). The 16S rRNA gene sequences retrieved were curated following the Ribosomal Data Project pipeline by removing primers and barcodes and filtering low quality and length reads, including sequences with ambiguity codes (Cole et al., 2014). The trimmed sequences were then taxonomically classified using the automatic software pipeline SILVAngs^1^ (Quast et al., 2013). The 16S RNA pyrolibraries were deposited in the European Nucleotide Archive (ENA) under study accession PRJEB14705 with the following run access numbers: ERS1237061 – ERS1237071.

Community composition was analyzed according to the initial contribution at Phyla and Order taxonomic levels as abundant (>0.5%), semi-rare (0.1–0.5%), and rare (<0.1%) in the total number of sequences retrieved from each pyrolibrary (Pedrós-Alió, 2012). Diversity indexes were estimated using Past 3 (Hammer et al., 2001). We used sequence contributions as percentage per pyrolibrary and not total numbers in our analyses and figures in order to normalize different library sizes. However, we also evaluated the potential effects of variation in pyrolibrary size by subsampling pyrolibraries using the subsample routine in MOTHUR to reflect the lowest number of reads encountered (1,100) and then re-analyzed our data as mentioned before.

The response of active bacteria to light treatments during hours of higher irradiances was estimated using the differences of the percentage contribution of a given order (taxonomical classification according to SILVAngs) between treatment pyrolibraries (py), considering the influence of UV (PA-PAR) relative to the effect of solar radiation from the full sun treatment (FS) according to the following equation:

$$\mathrm{Response}\;\mathrm{to}\;{\mathrm{UVR}}_{\;[\mathrm{Order}\%]}\;=\frac{Order_{\left[\%PApy\right]}\;-\;Order_{\left[\%PARpy\right]}}{Order_{\left[\%FSpy\right]}}$$

The response of the active orders to solar radiation (FS) compared to the dark treatment standardized by the initial community was also determined, considering the following equation.

$$\mathrm{Response}\;{\mathrm{FS}}_{\;[\mathrm{Order}\%]}\;=\frac{{\mathrm{Order}}_{\left[\%\;\mathrm{FSpy}\right]}{-Order}_{\left[\%\mathrm{Darkpy}\right]}}{({\mathrm{Order}}_{\left[\%Initial-in\;\mathrm{situ}\;\mathrm{py}\right]}}$$

A positive result was interpreted as stimulation, and negative results as inhibition for each of the active Orders examined based on their percentage of contribution to the pyrolibraries analyzed.

Clustering analyses were computed using available statistical software R 2.15.3 packages using the Manhattan index of distance and average clustering method. To evaluate confidence in the clustering results, bootstrap and an approximately unbiased (AU) *P*-value was computed after re-sampling 1000 times. The AU *P*-value was estimated because is less biased than the bootstrap probability value computed by ordinary bootstrapping (Suzuki and Shimodaira, 2013).

In order to visualize the overall shifts in the community structure of the active bacteria associated with both time and the different experimental treatments we conducted a Principal Coordinates Analysis (PCoA) based on a Bray Curtis similarity matrix of square-root transformed data of the relative contribution of the different active bacterial orders using the software PRIMER (7.0.11) with the PERMANOVA add on (Anderson et al., 2008). This form of ordination allows a visual representation of relative similarity in overall community structure where those markers found closer together are more similar.

In order to examine the relationship between overall shifts in community structure and the various abiotic variables measured during the experiment (water temperature; phosphate, nitrate, and nitrite concentration) we calculated the Pearson correlation between the various environmental variables (data log_10_ transformed and normalized) and Axis 1 and 2 of the PCo. We overlaid vectors of those variables with correlation values >0.2 to the PCo ordination allowing the main patterns between shifts in community structure and abiotic conditions to be depicted.

We used Spearman rank correlations (Statistica 7) to evaluate potential associations between variables measured during the experiments (temperature, solar dose, nutrient rates of change) and active Phyla shifts through the experiments.

## Results

### Physical and Chemical Changes Observed during the Experiment

We recorded extreme shifts in solar radiation through the experiment. Very high radiation levels (703 W m^-2^) were recorded just after sunrise, reaching 1,125 W m^-2^ at noon, 708 W m^2^ in mid-afternoon and falling to 54 W m^-2^ just prior to sunset (**Table 1**). The water from the spring was characterized as freshwater according to *in situ* measures of conductivity, showing slight changes through the incubation treatments during sampling (610–620 μS cm^-1^). Temperature was variable through the day, shifting from 14.3°C at dawn to 16–17°C between 11:30–15:30, and then falling to ca. 13°C at dusk (**Table 1**). In addition, bottle covering did not influence the temperature inside the bottles. Oxygen was also variable during the incubation, but this variability (3.9–5.7 mg L^-1^) was expected considering the mixing of water held in the bottles during subsampling and the shifts in temperature we showed. Spearman correlation analyses indicated that temperature was significantly correlated with total radiation (*r*_s_ = 0.71, *p* = 0.006) and doses (*r*_s_ = -0.64, *p* = 0.03), but no significant correlation was observed with nutrient concentration.

**Table 1:** Physical and chemical variables determined *in situ* (i.e., at the start of the experiment) and within the various treatments and incubation times.

Treatment (Time h)	Total Radiation W m^-2^ (Dose, KJ m^-2^)^∗^	Oxygen mg L^-1^	Conductivity μS cm^-2^	Temperature °C
*In situ* (9:30)	703 (3,610)	5.9	612	14.3
*In situ* (11:30)	1,125 (10,446)			
Dark (11:30)		3.9	612	16.2
PAR (11:30)		4.3	616	16.6
PA (11:30)		4.2	610	17.0
FS (11:30)		4.7	612	17.3
*In situ* (15:30)	708 (25,183)			
Dark (15:30)		4.8	612	17.0
PAR (15:30)		4.7	618	16.1
PA (15:30)		4.5	607	16.3
FS (15:30)		4.7	612	17.3
*In situ* (17:30)	54 (28,668)			
Dark (17:30)		5.47	610	12.0
PAR (17:30)		4.96	620	13.4
PA (17:30)		4.95	620	13.7
FS (17:30)		5.22	610	13.3

*Treatments acronyms: FS (full sun treatment between 280–700 nm), Dark (cover by aluminum foil and black plastic bags), PAR (400–700 nm, Photosynthetically Active Radiation), and PA (320–700 nm, UVR and PAR). ^∗^Radiation and Integrated Dose were determined at ground level *in situ*, whereas all the other parameters were measured inside the bottles during subsampling.*

### Nutrient Variability and Estimated Rates of Change through Time during the Incubation at the Different Treatments

**FIGURE 1:**
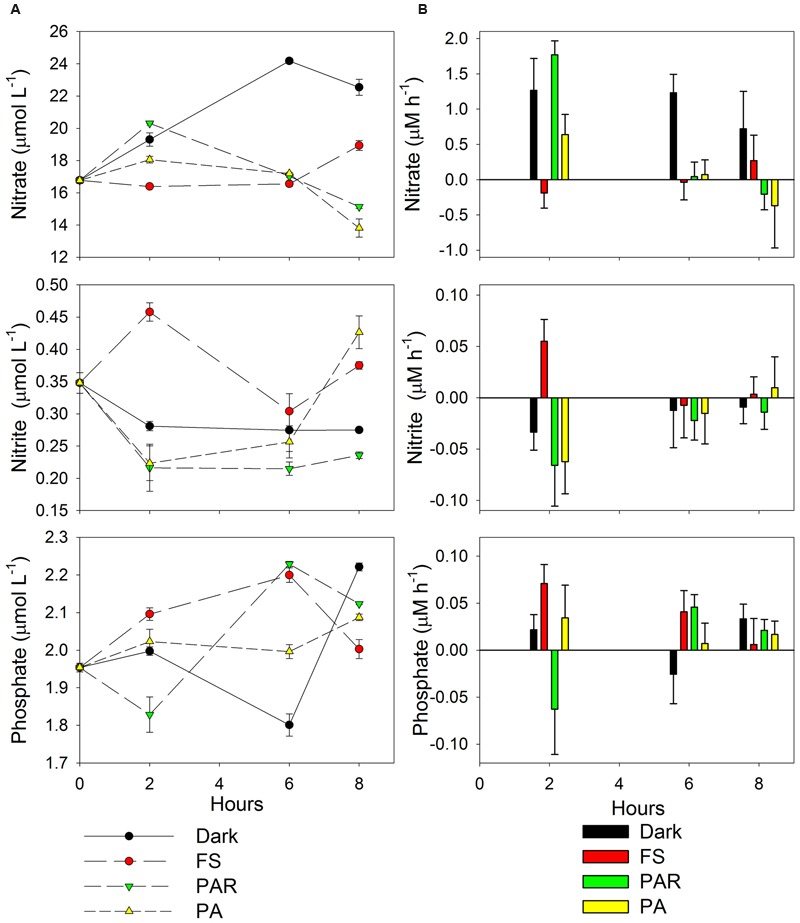
**(A)** Nitrate, nitrite and phosphate variability through the incubation and treatments. **(B)** Changes in nutrient concentration through time (μM h^-1^) obtained by end point determination approach. The rates were estimated from the different incubation treatments, considering the differences between the hours at 2, 6, and 8 h (11:30, 15:30, and 17:30 h) compared with *in situ* nutrient concentrations as initial time (9:30 h). Treatments acronyms: FS (full sun treatment between 280 nm–700 nm), Dark (cover by aluminum foil and black plastic bags), PAR (400–700 nm, Photosynthetically Active Radiation), and PA (320–700 nm, UVR and PAR).

The concentrations of nitrate and phosphate *in situ* were high compared to nitrite (**Figure 1A**) and also varied through the incubation and treatments, ranging between 13.8–24.2, 1.8–2.2, and 0.22–0.46 μmol L^-1^, respectively. A higher accumulation of nitrate through incubation time was observed mainly in dark treatments (0.72–1.27 μM h^-1^) followed by PAR and PA (11:30 h, 0.64–1.77 μM h^-1^), whereas a lower accumulation or consumption was observed in the rest of the treatments and incubation times (**Figure 1B**). Conversely, nitrite was largely consumed, and only accumulated under the FS (11:30 and 17:30 h) and PA (17:30 h) treatments. In contrast to nitrite, phosphate accumulated in almost all treatments except for PAR (11:30 h) and Dark (15:30 h).

Spearman’s correlation showed a negative relationship between the rates of change of nitrate and nitrite through the experiment (*r*_s_ = -0.62, *p* = 0.03), supporting that when nitrate accumulate, nitrite was mainly consumed.

### Active Bacteria Composition and Their Response during the Experiment Evolution

**FIGURE 2:**
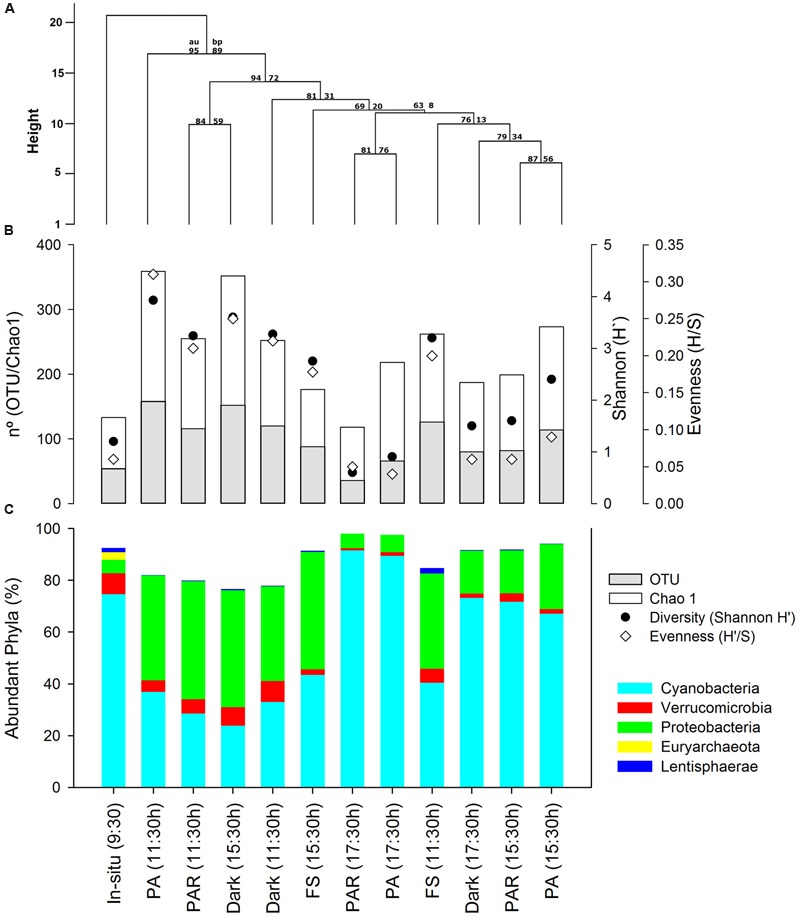
**(A)** Clustering of active bacteria community structure based on Manhattan distance and average clustering method, also AU (approximately unbiased *P*-value) and bootstrap values estimated using 1000 resampling are also depicted, **(B)** Abundant Phyla contribution (%) presented in the same order as clustering analyses results, and **(C)** plot showing richness, Chao1, Diversity (Shannon H′), and Evenness from the active bacterial community estimated from normalized by resizing pyrolibraries to 1,100 classified sequences.

A total of 33,939 sequences (average length of 514 bp) were analyzed, ranging between 1,101 and 7,664 sequences for each pyrolibraries (see details in **Supplementary Table S1**). The largest number of sequences reflected samples from the *in situ* pyrolibrary at 9:30 h (7,664 sequences), as well as the PAR (5,416) and PA (4,297) treatments from 17:30 h. In general, the pyrolibraries displayed a good coverage (0.94–0.99 based on expected versus observed richness), as supported by rarefaction curves (**Supplementary Figure S2**). These curves also indicated that the abundance of active classified bacterial OTUs (with the exception of the PA and PAR treatments from 17:30 h) were higher in almost all the treatments and times of incubation compared with *in situ* pyrolibraries. In addition, the cluster analysis based on the phyla contribution in the different pyrolibraries (**Figure 2A**) indicates that the *in situ* active bacteria community was most dissimilar to those seen in the various treatments. Furthermore, the active bacterial community was grouped by incubation time (11:30 h versus a 15:30 h and 17:30 h sub-clusters), with a further differentiation between PA (11:30 h) and FS (15:30 h).

More evidence for differences in bacterial communities associated with the different treatments was provided by the analysis of diversity and evenness indices (**Supplementary Table S1**; **Figure 2B**) Initial values of H′ (1.2–1.4) and H′/S (0.03–0.06) recorded from the *in situ* sample at the start of the experiment (09:30 h) showed a marked increase (H′3.2–3.9; H′/S 0.20–0.3), during hours of high radiation and temperature (11:30 h) particularly in the PA pyrolibrary, and then decreased again during the afternoon, including analyses in both the non and resized (∼1,100 sequences) pyrolibraries. Resized pyrolibraries presented negligible effects on the contribution of abundant and semi-rare phyla, however, many rare phyla were lost when libraries were resized. Therefore, subsequent analyses of active bacteria composition were conducted on percentages contribution data from non-resized pyrolibraries.

The initial composition of the *in situ* bacterial community (i.e., at the start of the experiment; 9:30 h) was mostly composed of the following phyla; Cyanobacteria (75%), Verrucomicrobia (6%), Proteobacteria (5%), Lentisphaerae (2%), with a higher number of phyla associated with the low frequency groups semi-rare and rare, some associated with the Candidate Radiation Phyla (CPR; Brown et al., 2015), including the Parcubacteria, OP3, Gracilibacteria, TM6, and SR1 (**Supplementary Table S1**; **Figure 2C**). Also, Euryarchaeota sequences were retrieved in the *in situ* pyrolibrary associated with methanogens, representing 3% of the total sequences (**Figure 2C**). Incubation under the different experimental treatments resulted in changes in active bacterial community structure, characterized mainly by a decrease in the contribution of Cyanobacteria in the 11:30 h light and dark treatments, and an increase in this phyla during the afternoon (17:30 h). In general, an opposite pattern was apparent for the Proteobacteria and Lentisphaerae, and other semi-rare and rare phyla (**Figure 2C**).

**FIGURE 3:**
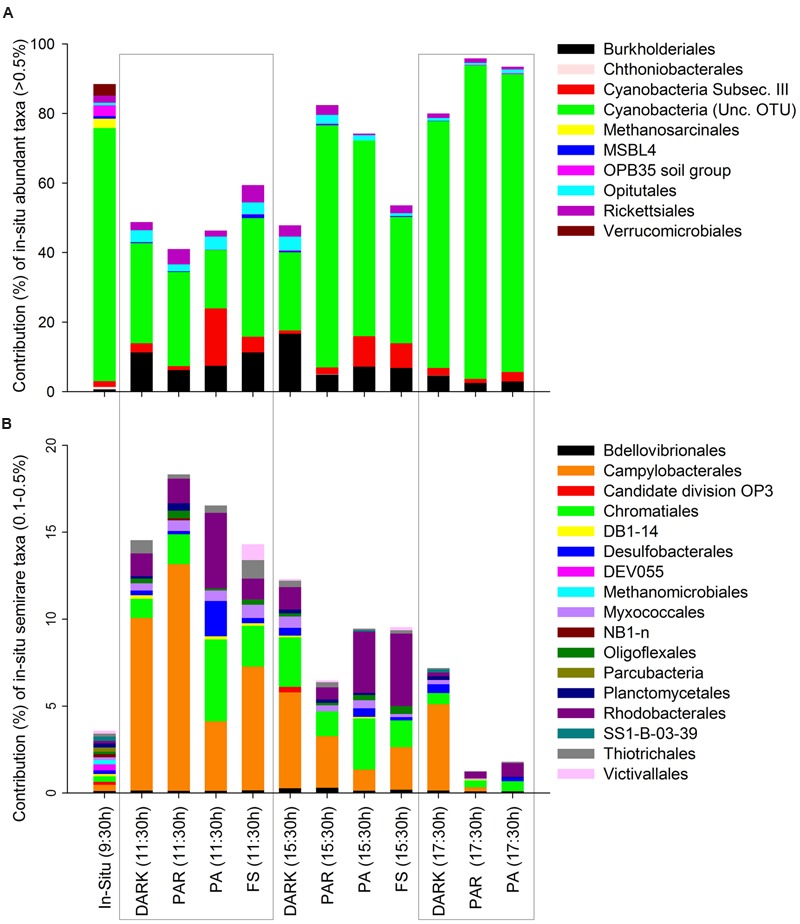
**Contribution of active Orders classified according to their initial *in situ* contribution as abundant (A)** and semi-rare **(B)** in percentages (>0.5 and 0.1–0.5% of pyrolibraries, respectively), and their changes through the time of the experiment at the different treatments.

**FIGURE 4:**
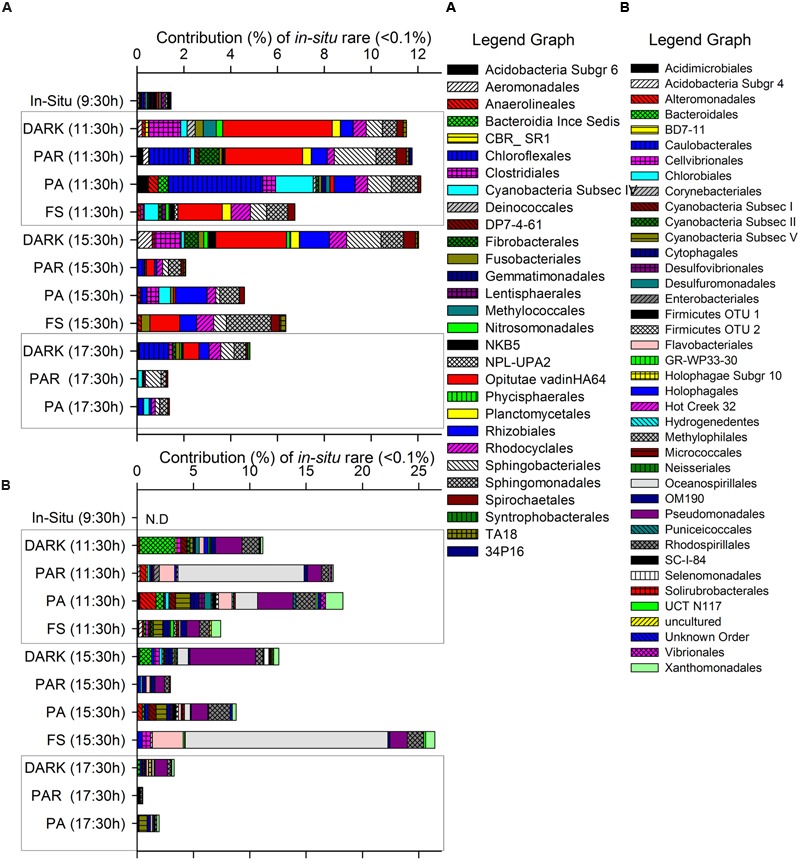
**Contribution of specific active taxa (mainly Orders when available) classified as rare according to their initial *in situ* pyrolibrary contribution as groups detected >0-<0.1% (A)** or undetected 0% **(B)** and the changes determined during the incubation through the time of the experiment at the different treatments.

The specific composition of the active microbial community at the taxonomic level of order (or lowest taxon level available for some groups) is shown according to their initial contribution (*in situ*) as abundant (**Figure 3A**), semi-rare (**Figure 3B**), and rare groups (**Figure 4**). The contribution of abundant and semi-rare taxa in the different treatments and over time relative to the *in situ* data indicates that some groups were only detected *in situ*, and not during experimental treatments, such as, OPB35 soil, uncultured Verrucomicrobiales, Chthoniobacterales, DEV055, SS1-B-03-39, DB1-14, NB1-n (Tenericutes), and also the Euryarchaea Methanosarcinales and Methanomicrobiales (**Figure 3**). Some groups presented noticeable temporal patterns, such as Cyanobacteria (from an uncultured OTU) characterized by a reduction in their contribution to pyrolibraries from the first 2 h of incubation in the different treatments, but largely in PA (11:30 h), followed by an increase in the afternoon across treatments (**Figure 3A**). In contrast, Cyanobacteria from Subsection III, such as *Microcoleus, Oscillatoria, and Phormidium* increased their activity at PA (11:30 h) and also in the afternoon, whereas other bacteria such as Burkholderiales, Opitutales, and Rickettsiales were characterized by decrease at the afternoon treatments (**Figure 3A**).

In general, semi-rare groups increased significantly compared to their initial contribution to the *in situ* sample at the start of the experiment, making individual high percentage contributions to the pyrolibraries, e.g., Candidate division OP3, Campylobacterales, Chromatiales, Rhodobacterales (**Figure 3B**), mainly in the first 2 h of incubation across all treatments, but specifically in the PA treatment at 11:30 h.

**Figure 4** shows those groups that were initially rare (**Figure 4A**: <0.1% of contribution to the pyrolibrary) or even undetected (**Figure 4B**) at the start of the experiment in the *in situ* sample, and that showed an increased contribution to the pyrolibraries over the incubation period, suggesting that these groups presented a low activity or were inactive at the beginning of the experiment. This included some phototrophic and non-phototrophic bacteria, such as Flavobacteriales, Sphingobacteriales, Chloroflexales, Cyanobacteria Subsection IV (Nodularia, Nostoc, Trichormus, Calothrix, Rivularia, Uncultured OTU) and V (Stigonema), Fibrobacterales, Oceanospirillales, Pseudomonadales. Other taxa that increased over time were related to alternative light-harvesting strategies (Rhodocyclales, Chloroflexales), as well as other metabolic pathways (Methylococcales, Nitrosomonadales, Rhizobiales, Anaerolineales, Syntrophobacterales; **Figures 4A,B**).

**FIGURE 5:**
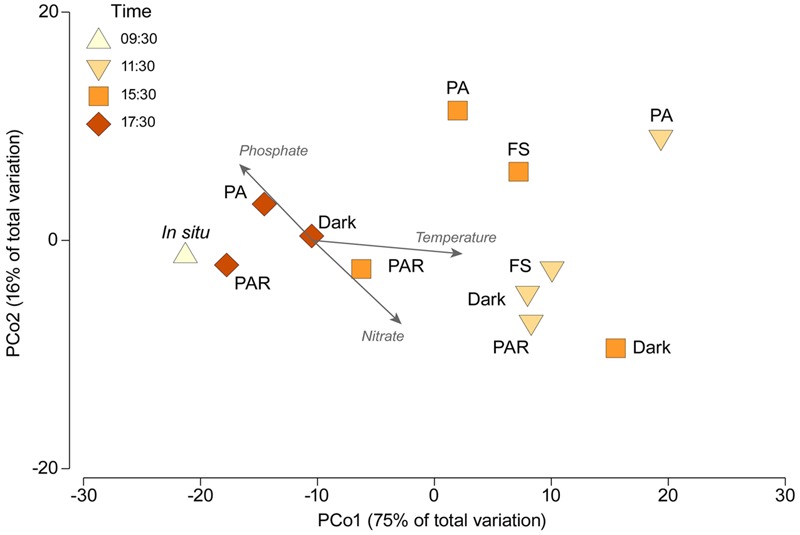
**Principal coordinates analysis (PCoA) ordination showing variation in active bacteria community structure associated with the different treatments and time periods.** Vectors show those abiotic factors shown to be correlated (*r* > 0.2) with PC Axis 1 or 2. Treatments acronyms: FS (full sun treatment between 280–700 nm), Dark (cover by aluminum foil and black plastic bags), PAR (400–700 nm, Photosynthetically Active Radiation), and PA (320–700 nm, UVR and PAR).

A PCoA ordination based on Bray–Curtis distance is presented in **Figure 5**. The results of this analyses showed that the greatest dispersion in community structure were associated with the samples collected at 15:30 and PA (11:30 h), with the other treatments generally clustering close together in each time period. Vectors showing correlations with environmental variables measured at the same time reveal that Water temperature was positively correlated (*r* = 0.77) with Axis 1 (75% of variation), as was nitrate concentrations (*r* = 0.46). Phosphate concentration correlations was negatively correlated with Axis 1 (*r* = -0.38). Axis 2 (16% of variation) was negatively correlated (*r* = -0.44) with nitrate concentrations and positively correlated (*r* = 0.4) with phosphate concentrations. Vectors are not shown for nitrite or the total radiation dose as both variables showed little evidence for correlation (*r* < 0.2).

Significant correlations were found between the rate of change in nitrate concentrations and the following active bacteria Phyla: Acidobacteria (*r*_s_ = 0.61, *p* = 0.04), Bacteroidetes (*r*_s_= 0.75, *p* = 0.01), Chloroflexi (*r*_s_ = 0.62, *p* = 0.04), Cyanobacteria (*r*_s_= -0.75, *p* = 0.01), Firmicutes (*r*_s_= 0.78, *p* = 0.004), TM6 (*r*_s_ = 0.61, *p* = 0.05), Verrucomicrobia (*r*_s_ = 0.71, *p* = 0.01). Also correlations were found between the rate of change of nitrite concentration with Tenericutes (*r*_s_= -0.66, *p* = 0.03) and between phosphate and TM6 (*r*_s_ = -0.67, *p* = 0.02).

### Light Quality Effect on Active Bacterial Community

The responses of the active bacterial community to different light treatments after 2 h of incubation are shown in **Table 2**. In general, many orders were stimulated by light treatments (positive values) under both UVR and FS or one of the each sun light quality (**Table 2**). Some of the bacteria orders that were stimulated by both UVR and FS, e.g., Chromatiales, Cyanobacteria subsection III (*Microcoleus, Oscillatoria*) amd IV (*Nodularia*, uncultured OTU), Desulfobacterales, Rhodocyclales, Sphingomonadales, Thiotrichales. A limited number of groups were apparently inhibited by UVR and FS, such as Campylobacterales, Opitutae vadinHA64, Sphingobacteriales. Conversely, some groups displayed a stimulatory response to UVR but were inhibited by the FS treatment including Burkholderiales, Clostridiales, Opitutales, Rhodobacterales, while some bacteria showed an opposite reaction including Bdellovibrionales, Cyanobacteria (Unc. OTU), Myxococcales, Oligoglexales, Rickettsiales, Spirochaetales (**Table 2**).

**Table 2:** Characterizing the response of bacterial orders present (percentages) *in situ* and in all the different treatments and their response to UVR and FS, in terms of whether they were stimulated (positive values) or inhibited (negative values) under experimental treatments at hours of high irradiation (11:30 h).

	Percentage of contribution					Stimulatory or Inhibitory response	
Bacterial order	*In situ*	DARK	FS	PAR	PA	UVR	FS
Bdellovibrionales	0.12	0.14	0.15	0.12	0.12	-0.02	0.12
Burkholderiales	0.64	11.30	11.28	6.21	7.40	0.11	-0.04
Campylobacterales	0.35	9.92	7.12	13.04	4.00	-1.27	-7.95
Chromatiales	0.31	1.10	2.35	1.72	4.72	1.28	3.96
Clostridiales	0.08	1.38	0.15	0.12	0.54	2.74	-15.63
Cyanobacteria (Unc. OTU)	72.81	28.81	34.07	27.12	16.78	-0.30	0.07
Cyanobacteria Subsec III	1.67	2.62	4.54	1.11	16.54	3.40	1.15
Cyanobacteria Subsec IV	0.07	0.28	0.61	0.18	1.61	2.36	5.04
Desulfobacterales	0.21	0.28	0.30	0.18	2.03	6.09	0.13
Myxococcales	0.16	0.41	0.76	0.62	0.60	-0.02	2.19
Oligoflexales	0.13	0.28	0.30	0.43	0.12	-1.03	0.21
Opitutae vadinHA64	0.08	4.69	1.89	3.32	0.18	-1.66	-35.59
Opitutales	0.73	3.45	3.33	1.97	3.76	0.54	-0.16
Rhodobacterales	0.14	1.31	1.21	1.41	4.36	2.43	-0.68
Rhodocyclales	0.09	0.55	0.83	0.31	0.54	0.28	3.07
Rickettsiales	2.08	2.34	5.00	4.43	1.73	-0.54	1.28
Sphingobacteriales	0.05	0.69	0.68	1.78	1.01	-1.13	-0.15
Sphingomonadales	0.09	0.62	0.91	0.86	1.13	0.30	3.15
Spirochaetales	0.03	0.28	0.30	0.43	0.12	-1.03	1.03
Thiotrichales	0.17	0.76	1.06	0.25	0.42	0.16	1.77

*See “Materials and Methods” section for more details.*

## Discussion

### Active Microbial Community Phyla of a High-Altitude Wetland Spring Site

Cyanobacteria, Verrucomicrobia, Proteobacteria, and also the non-specifically amplified Euryarchaeota (Methanosarcinales) were the most active phyla detected *in situ*. These phyla are commonly detected in DNA extracted from the water of high-altitude ecosystems from the central Andes (Albarracin et al., 2015), including previous diversity studies using 16S RNA gene cloning for Salar de Huasco (Dorador et al., 2008b, 2009, 2013). Thus, our findings based on 16S rRNA pyrosequencing supports previous studies and given that our results originate from cDNA indicate that wide diversity of phyla previously identified are not only present, but also potentially active. Moreover, our results allowed us to identify a high number of other potentially active phyla, including Lentisphaerae and also others from the recently described Candidate Phyla Radiation (Brown et al., 2015), e.g., Parcubacteria, Gracilibacteria, OP3 (Omnitrophica), TM6, SR1, and groups found at low, semi-rare (<0.5%) and rare (<0.1%) frequencies.

### Potential Biases Associated with Microbial Community Changes during the Experiment

The *in situ* active bacterial community displayed marked changes associated with both time and experimental treatments: these shifts are clearly displayed through both clustering and PCoA analyses (**Figures 2** and **5**). We are confident that these patterns largely reflect changes in environmental conditions and responses to our experimental treatments, but recognize the potential for experimental artifacts to have influenced our results. The incubation setup we used could have potentially inhibited the metabolism of some anaerobic active phyla *in situ*. For example, active Parcubacteria and Euryarchaeota were detected *in situ* but not in incubated samples (**Figure 2**; **Supplementary Table S1**). The presence of these groups *in situ* could originate from sediment particles, since they require anoxic conditions for their metabolism, conditions not expected in the water based on our dissolved oxygen concentration values (**Table 1**). Parcubacteria have been detected in the metagenome of widespread anoxic or oxygen-limited ecosystems and also from artificial environments, probably reflecting a symbiotic lifestyle (Rinke et al., 2013; Nelson and Stegen, 2015). A similar situation was expected to occur for Euryarchaeota sequences related to known anaerobic methanogens (Methanomicrobiales and Methanosarcinales, **Figure 3**), which were previously reported as a prevalent archaea from sediments sampled from the current study site, H0 (Dorador et al., 2013). In addition, bottle effects due to enclosure have been reported to result in significant changes in microbial composition (Schafer et al., 2000; Piccini et al., 2009). They have also been associated with changes in gene transcription patterns, including the enhancement of gene transcripts associated with the detection and transport of organic and inorganic extracellular solutes in bioreactor metatranscriptomic studies, compared with *in situ* samples from oxygen minimum zones (Stewart et al., 2012). Our experimental design did not include treatment replicates, limiting our scope for interpretation and the capacity to exclude potential stochastic effects.

### Active Bacterial Dynamics, the Impact of Solar Radiation and Potential Effects on Nutrient Recycling

Across the different incubation periods, but most notable at noon, variation in the community structure of active bacteria (based on cDNA) was higher in light (PA at 11:30 h) compared to *in situ* and afternoon-dusk treatments (**Figures 2A** and **5**). Moreover, at noon, when solar radiation and water temperature was highest, rates of change in nutrient concentrations were highest and also revealed shifts in both production and consumption (**Figure 1B**).

Cyanobacteria (Unc. OTU) were one of the most abundant phylum *in situ*, but displayed considerable changes in terms of their relative contribution to the pyrolibraries during the experimental incubation. These shifts were characterized by a decline in all treatments at 11:30 h compared to *in situ* data, (most apparent in the PA treatment), followed by an increasing trend in samples from the afternoon (**Figure 2**; **Supplementary Table S1**). This result suggests an inhibitory effect of light on the abundant Cyanobacteria (Unc. OTU) when potential activity during hours with elevated radiation resulted in an inhibitory response to UVR (**Table 2**). Our data also indicate the potential for the Cyanobacteria (Unc. OTU) to demonstrate a rapid recovery once radiation levels fall in the afternoon. In general, UVR can cause substantial damage to many cellular components, including production of photoproducts in DNA and photooxidation of proteins and unsaturated lipids in cell membranes (Ravanat et al., 2001). In photosynthetic microorganisms, UVR can also produce photodamage and repair inhibition of photosystem II, and destruction of light-harvesting phycobiliproteins, due to an increase in oxidative stress (Nishiyama et al., 2005).

However, photosynthetic microorganisms have developed a number of physiological and biochemical strategies to cope with, and to adapt to the detrimental effects of UVR (Castenholz, 2004). Phototrophic bacteria minimize and prevent photo-oxidative damage through the production of enzymes including glutathione peroxidase, thioredoxins, and superoxide dismutase (Castenholz, 2004; Zeller et al., 2005). Moreover, carotenoids, amino acid derivates, and osmoprotectants can also play an important role as UV-absorbing compounds and reactive oxygen species scavengers: photolyases and other protective agents photo-reactivated via UVA and PAR wavelengths can also act as repair mechanisms (Rastogi et al., 2010). These mechanisms could be expected to be present in the Cyanobacteria subsection III (*Microcoleus, Oscillatoria*) and IV (*Nodularia*, Uncultured OTUs), both of which showed a stimulatory response to UVR and FS, and also in other phototrophic (e.g., Chromatiales) and non-phototrophic bacteria (e.g., Desulfobacterales) during periods of high radiation (**Table 2**). These groups were expected to reveal higher activity (based on 16S rRNA) in response to UVR compared to other pyrolibraries (see positive results for UVR for some bacteria orders in **Table 2**), since our data reflect RNA and ribosomes, which are needed for growth and de novo enzyme production. Photo-reactivation has been described in several Cyanobacteria species, e.g., *Synechocystis* sp. PCC 6803 (Vass et al., 2013), *Anabaena* sp., PCC 7937 (Singh et al., 2013), and *Anacystis nidulans* (Eker et al., 1990). Specifically, a possible role of pterins as a protective agent against UVA radiation has also been suggested in species belonging to Cyanobacteria subsec III, such as *Oscillatoria* sp., as exposure to UVA caused a rapid onset of synthesis and massive accumulation of BH4 biopterin glucoside in irradiated cells (Wachi et al., 1995). Moreover, mycosporine-like amino acids have been described as a sunscreen protection strategy in Cyanobacteria, including subsec III *Microcoleus* (Pattanaik et al., 2008), a genus that in our results was activated associated by exposure to UVA (PA treatments). Little information is available regarding the response to light quality effects for the other phototrophic and non-phototrophic bacteria detected here. However, our results support previous findings of a photo-stimulation and differential tolerance to UVR and PAR in Alpha- and Gammaproteobacteria from marine ecosystems (Alonso-Sáez et al., 2006; Ruiz-Gonzalez et al., 2012, 2013). Future studies using more specific approaches, such as comparison of RNA vs. DNA through qPCR, should consider evaluating the specific responses of different bacterial groups shown here to react to UVR and/or FS, as employed in some studies based on natural community dynamics (Campbell et al., 2009; Levipan et al., 2014).

The direct negative influence of solar radiation on the abundant active Cyanobacteria (Unc. OTU) found at site H0 generated cascading cycling effects on low frequency and rare bacteria throughout the incubation period (hours), increasing their contribution to the active bacteria community in treatments associated with high solar radiation and UVR (PA) (**Figures 3** and **4**). The existence of such changes were also supported by changes in diversity and evenness indices calculated for the active microbial community during our experiment (**Figure 2**; **Supplementary Table S1**) using both uncorrected pyrolibraries and those corrected for variation in sequence abundance. We recorded a greater than twofold increase in Shannon’s H′ from initial values of 1.2–1.4 to a maximum of 3.9 in the PA treatment at 11:30 h, a period of high radiation and temperature, followed by a decline during the afternoon to H′ values of 0.5–1.5. The high bacterial diversity was associated with a reduced contribution by Cyanobacteria, and an increase contribution from Proteobacteria, as well as from other semi-rare and rare bacteria (**Figures 3B** and **4A,B**), including some groups not detected *in situ*, but which were activated at hours of high radiation, largely in the PA treatment (**Figure 4B**).

Previous reports based on studies using a range of methodological approaches and from different ecosystems have indicated that rare bacterial groups respond to changes in their environment by increasing their abundance relative to the rest of the microbial community across different timescales. For example, previously rare bacterial communities were found to activate and increase their abundance after dry soils were moistened, potentially reflecting resuscitation after dormancy, based on experimental enrichment of heavy water and DNA stable-isotope probing and identification of “newly active rare members” by 16S rDNA (Aanderud et al., 2015). In addition, temporal patterns could also influence patterns of apparent predominance between members of the microbial community. This has been seen in studies reporting cycling in relative abundance from rare, through to low frequency and then to abundant in coastal (Campbell et al., 2009, 2011) and oligotrophic (Vergin et al., 2013) marine environments, as well as in lake microbial communities responding to natural and forced mixing events (Shade et al., 2014). A recent review (Shade and Gilbert, 2015) identified some of these taxa as being “conditionally rare” and discussed the need for further studies to determine their role in the dynamics of microbial communities. Here, at a single site, we found that the cycling of the microbial community within a single day could be related to both direct and indirect effects of solar radiation. Direct effects likely include photoinhibition of phototrophic activities in Cyanobacteria during hours of high radiation (discussed above), while indirect effects likely include the formation of niches for other members of the microbial community, including groups with different lifestyles, such as heterotrophic and chemolithoautotrophic bacteria, as reported from marine ecosystems (Alonso-Sáez et al., 2006; Ruiz-Gonzalez et al., 2013).

During our experiments, a higher accumulation of nitrate (except for FS) and phosphate (except for PAR) and consumption of nitrite (except for FS) were recorded at noon when Cyanobacteria were light-inhibited. Reduced accumulation (low or even negative rates of change) was observed toward the afternoon, when Cyanobacteria activity recovered (**Figure 1**). An association between these two factors is supported by a negative correlation between the relative abundance of Cyanobacteria with the rate of change in nitrate concentrations. Moreover, the accumulation of nitrate and the loss of nitrite during the first 2 h of the incubation and later (4 h and 6 h, except for FS and PA-PAR, respectively) potentially reflect increased activity by nitrifying bacteria (**Figure 1**). Both ammonia and nitrite oxidizing bacteria from the genera *Nitrosomonas* and *Nitrospira*, respectively, were detected during our experiment (**Figure 4**; **Supplementary Table S1**). *Nitrosomonas* was detected both *in situ* and from experimental (mainly dark) treatments, while *Nitrosomonas* and *Nitrospira* were both detected in the afternoon dark treatment (17:30 h), coinciding with the accumulation of nitrate and nitrite consumption (**Figure 1**). Ammonia-oxidizing bacteria are not strong competitors for ammonia compared to either phototrophic organisms or their ammonia-oxidizing archaeal counterparts (Martens-Habbena et al., 2009). As such, they potentially favored conditions when Cyanobacteria were less active, such as found in the dark treatment through the experiment and under certain light treatments. However, despite these taxa being active, their level of activity was reduced in FS compared to dark treatments. The order Nitrosomonadales was not detected in PAR and PA treatments during the period of high radiation, during when nitrite accumulation was recorded in FS (**Figure 4A**). This result supports previous reports of photoinhibition effects on nitrifying communities, including ammonia-oxidizing bacteria and archaea, and nitrite oxidizers (Olson, 1981; Ward, 1985; Guerrero and Jones, 1996; French et al., 2012; Merbt et al., 2012). As well as nitrifying taxa, many bacteria could potentially have played a role in the nutrient fluxes observed during our experiments, including phototrophic, heterotrophic, and chemoautotrophic bacteria. The relative abundance of several bacterial taxa including Acidobacteria, Bacteroidetes, Chloroflexi, Firmicutes, Verrucomicrobia were all significantly correlated with nitrate, and with the community structure of active bacteria (**Figure 5**). Many of these groups were originally inactive or present at low activity *in situ*, but increased their activity during the experiment, particularly in dark treatments, such as Bacteroidetes and Verrucomicrobia, which are heterotrophic groups that can potentially use nitrate to respire. This supports previous reports showing a significant reaction of Verrucomicrobia to higher nitrogen loads in lake mesocosm studies (Haukka et al., 2006). Moreover, changes in composition between Bacteroidetes and other bacteria such as Cytophaga and Proteobacteria have been associated with the presence of labile organic compounds originating from the photo-alteration of dissolved organic matter in coastal lagoon (Piccini et al., 2009) and marine environments (Ruiz-Gonzalez et al., 2013). Furthermore, Verrucomicrobia, Bacterioidetes, Planctomycetes and others have been related to Cyanobacteria temporal dynamics in an ephemeral lake based on bacterial network analyses (Woodhouse et al., 2016). In addition, many active bacteria detected in our work potentially contribute to nutrient shifts, including the Rhodocyclales, Campylobacterales as well as other taxa related with sulfur metabolism (Desulfobacterales, Chromatiales) in the different incubations.

Taken together, our results suggest that active bacterial communities in this high-altitude wetland respond rapidly, and differently to variation in solar radiation. Our data reveal that Cyanobacteria were clearly the most sensitive component of the community to elevated levels of solar radiation, but importantly revealed that they were resilient to the potential effects of photodamage, as seen by their rapid recovery later in the day. Variation in the Cyanobacteria was reflected by an increase in the total diversity of the active bacteria community, characterized by the increased contribution of abundant Proteobacteria, but also by increased contributions from both semi-rare and rare bacteria. In turn, changes in the structure of the active bacterial community associated with daily abiotic variability were shown to influence nutrient recycling rates, highlighting how abiotic variation affects potential ecosystem functioning.

## Author Contributions

VM and CD wrote the paper, participate in the sampling collection during the experiment, molecular, and nutrient analyses. KH designed the experiment setup, execute the experiment in the field, analyzed solar radiation data, contribute in paper writting. YE, CD, MH, and CH help in paper writting and field trip experiments. VP and CH assist in paper writting and data analyses. VM, CD, and KH are PIs of project grants supporting fieldtrip and data analyses.

## Conflict of Interest Statement

The authors declare that the research was conducted in the absence of any commercial or financial relationships that could be construed as a potential conflict of interest.

## Footnotes

1 https://www.arb-silva.de/

## Supplementary Material

FIGURE S1 **(A)** Photographs showing the experiment site and setup and **(B)** the map of the study area (Salar de Huasco, Chile).

FIGURE S2 **Rarefaction curves derived from classified active bacteria OTUs detected from the 16S rRNA pyrolibraries (cDNA) generated from samples collected *in situ* and the following experimental incubation treatment.** Treatments acronyms: FS (full sun treatment between 280–700 nm), Dark (cover by aluminum foil and black plastic bags), PAR (400–700 nm, Photosynthetically Active Radiation), and PA (320–700 nm, UVR and PAR).

TABLE S1 **Pyrolibraries information, including coverage (Chao1), ecological indexes (OTU and Shannon H′), and Phyla changes through the incubations depicted by gray gradients as abundant (>0.5%, semi-rare 0.5–0.1%, and rare <0.1%).** Treatments acronyms: FS (full sun treatment between 280–700 nm), Dark (cover by aluminum foil and black plastic bags), PAR (400–700 nm, Photosynthetically Active Radiation), and PA (320–700 nm, UVR and PAR).
