# Supplementary figures and images for: Bacterial Active Community Cycling in Response to Solar Radiation and Their Influence on Nutrient Changes in a High-Altitude Wetland
Source: Front Microbiol. 2016 Nov 17;7:1823. doi: 10.3389/fmicb.2016.01823 (PMC5112256; doi:10.3389/fmicb.2016.01823)

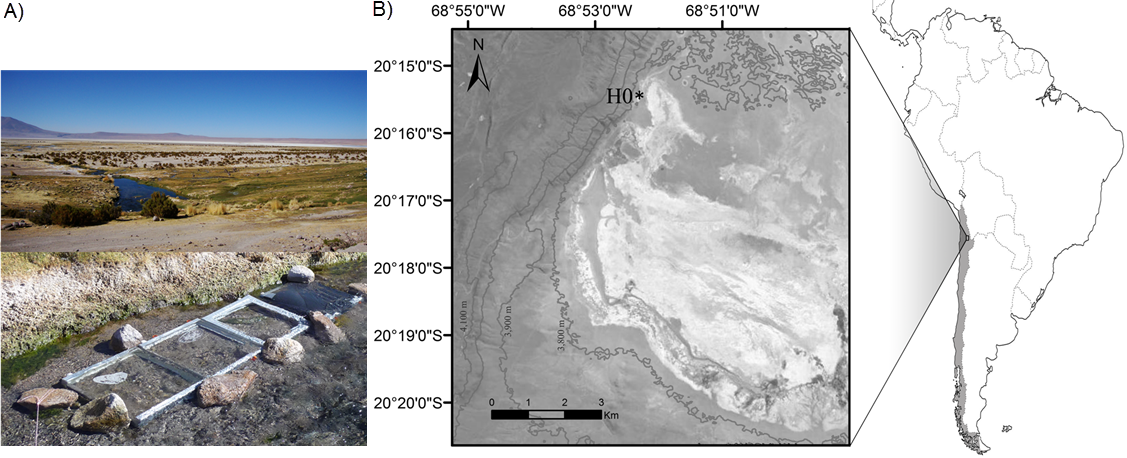

Supplement: FIGURE S1 — (A) Photographs showing the experiment site and setup and (B) the map of the study area (Salar de Huasco, Chile). [file Image_1.TIF]

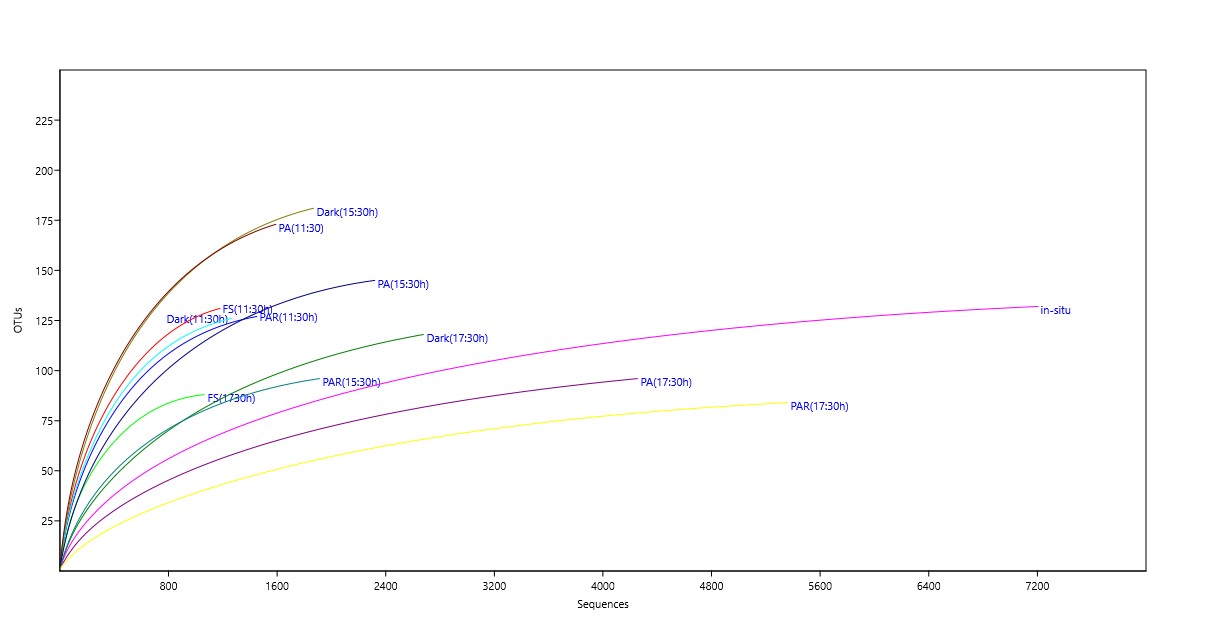

Supplement: FIGURE S2 — Rarefaction curves derived from classified active bacteria OTUs detected from the 16S rRNA pyrolibraries (cDNA) generated from samples collected in situ and the following experimental incubation treatment. Treatments acronyms: FS (full sun treatment between 280–700 nm), Dark (cover by aluminum foil and black plastic bags), PAR (400–700 nm, Photosynthetically Active Radiation), and PA (320–700 nm, UVR and PAR). [file Image_2.jpeg]
