# Supplementary material for: Bacterial Active Community Cycling in Response to Solar Radiation and Their Influence on Nutrient Changes in a High-Altitude Wetland
Source: Front Microbiol. 2016 Nov 17;7:1823. doi: 10.3389/fmicb.2016.01823 (PMC5112256; doi:10.3389/fmicb.2016.01823)
Supplement: TABLE S1 — Pyrolibraries information, including coverage (Chao1), ecological indexes (OTU and Shannon H′), and Phyla changes through the incubations depicted by gray gradients as abundant (>0.5%, semi-rare 0.5–0.1%, and rare <0.1%). Treatments acronyms: FS (full sun treatment between 280–700 nm), Dark (cover by aluminum foil and black plastic bags), PAR (400–700 nm, Photosynthetically Active Radiation), and PA (320–700 nm, UVR and PAR). [file Table_1.DOCX]

Table S1. Pyrolibraries information, richness (OTU, Chao1), diversity (H`) and evenness (H`/S) indexes and Phyla changes through the incubations depicted by grey gradients as abundant (>0.5%, semirare 0.5-0.1% and rare <0.1%). In parentheses associated to richness estimators and indexes are information obtain in a parallel analyses carried out after resizing the libraries for normalization purposes (1,100 sequences). *Results of phyla percentage contribution after resized to 1,100 sequences.

| **Treatment (Time h)** | **In-situ (9:30h)** | **Dark (11:30h)** | **PAR (11:30h)** | **PA (11:30h)** | **FS (11:30h)** | **Dark (15:30h)** | **PAR (15:30h)** | **PA (15:30h)** | **FS (15:30h)** | **Dark (17:30h)** | **PAR (17:30h)** | **PA (17:30h)** |
| --- | --- | --- | --- | --- | --- | --- | --- | --- | --- | --- | --- | --- |
| **Nº Sequences (% classified)** | 7664 (94) | 1451 (87) | 1626 (89) | 1675 (95) | 1321 (89) | 2178 (85) | 2027 (94) | 2383 (97) | 1101 (97) | 2800 (96) | 5416 (99) | 4297 (99) |
| **Max Length** | 540 | 513 | 519 | 513 | 514 | 516 | 517 | 513 | 506 | 521 | 521 | 506 |
| **OTU (resized)** | 132 (54) | 126 (120) | 127 (116) | 173 (158) | 131 (126) | 181 (152) | 96 (82) | 145 (114) | 88 (88) | 118 (80) | 84 (36) | 96 (66) |
| **Chao 1 (resized)** | 138 (79) | 131 (132) | 131 (139) | 182 (201) | 135 (136) | 189 (200) | 101 (117) | 149 (159) | 89 (89) | 132 (107) | 90 (82) | 112 (152) |
| **Shannon Index (resized)** | 1.4 (1.2) | 3.3 (3.3) | 3.2 (3.2) | 3.9 (3.9) | 3.3 (3.2) | 3.7 (3.6) | 1.6 (1.6) | 2.5 (2.4) | 2.8 (2.8) | 1.6 (1.5) | 0.6 (0.6) | 0.9 (0.9) |
| **Evenness (resized)** | 0.03 (0.06) | 0.21 (0.22) | 0.20 (0.21) | 0.29 (0.31) | 0.20 (0.20) | 0.23 (0.25) | 0.05 (0.06) | 0.08 (0.09) | 0.18 (0.18) | 0.04 (0.06)) | 0.02 (0.05) | 0.03 (0.04) |
| Cyanobacteria | 74.64 | 33.01 | 28.54 | 36.90 | 40.42 | 23.88 | 71.70 | 67.09 | 43.42 | 73.28 | 91.57 | 89.49 |
| Verrucomicrobia | 8.14 | 8.13 | 5.47 | 4.42 | 5.37 | 7.16 | 3.31 | 1.77 | 2.18 | 1.61 | 0.79 | 1.33 |
| Proteobacteria | 5.17 | 36.53 | 45.70 | 40.60 | 36.79 | 45.13 | 16.54 | 25.14 | 45.41 | 16.54 | 5.67 | 6.78 |
| Euryarchaeota | 2.94 | 0.00 | 0.00 | 0.00 | 0.00 | 0.00 | 0.00 | 0.00 | 0.00 | 0.00 | 0.00 | 0.00 |
| Lentisphaerae | 1.66 | 0.28 | 0.18 | 0.06 | 2.12 | 0.46 | 0.35 | 0.13 | 0.45 | 0.25 | 0.00 | 0.02 |
| *Cyanobacteria | 76.39 | 33.45 | 28.09 | 37.00 | 40.73 | 24.64 | 71.31 | 68.80 | 43.45 | 73.82 | 91.71 | 89.16 |
| *Verrucomicrobia | 6.38 | 7.55 | 6.09 | 4.73 | 5.36 | 7.91 | 3.37 | 1.46 | 2.18 | 1.45 | 0.91 | 2.00 |
| *Proteobacteria | 5.74 | 36.36 | 45.82 | 40.91 | 36.55 | 43.27 | 17.03 | 23.54 | 45.36 | 17.64 | 6.28 | 6.10 |
| *Euryarchaeota | 3.28 | 0.00 | 0.00 | 0.00 | 0.00 | 0.00 | 0.00 | 0.00 | 0.00 | 0.00 | 0.00 | 0.00 |
| *Lentisphaerae | 1.37 | 0.36 | 0.18 | 0.09 | 2.00 | 0.55 | 0.36 | 0.18 | 0.45 | 0.18 | 0.00 | 0.09 |
| **Planctomycetes** | 0.34 | 1.03 | 1.23 | 0.00 | 0.91 | 0.96 | 0.54 | 0.21 | 0.18 | 0.36 | 0.04 | 0.26 |
| **Parcubacteria** | 0.25 | 0.00 | 0.00 | 0.00 | 0.00 | 0.00 | 0.00 | 0.00 | 0.00 | 0.00 | 0.00 | 0.00 |
| **Tenericutes** | 0.18 | 0.00 | 0.12 | 0.48 | 0.00 | 0.00 | 0.00 | 0.00 | 0.00 | 0.00 | 0.00 | 0.00 |
| **Candidate division OP3** | 0.17 | 0.00 | 0.00 | 0.00 | 0.00 | 0.32 | 0.00 | 0.00 | 0.00 | 0.00 | 0.00 | 0.00 |
| **Chloroflexi** | 0.16 | 0.55 | 1.66 | 4.66 | 0.30 | 0.18 | 0.30 | 0.42 | 0.18 | 1.29 | 0.04 | 0.28 |
| **Firmicutes** | 0.10 | 1.38 | 0.25 | 1.19 | 0.15 | 1.65 | 0.10 | 1.01 | 0.00 | 0.21 | 0.00 | 0.02 |
| *Parcubacteria | 0.36 | 0.00 | 0.00 | 0.00 | 0.00 | 0.00 | 0.00 | 0.00 | 0.00 | 0.00 | 0.00 | 0.00 |
| *Candidate division OP3 | 0.18 | 0.00 | 0.00 | 0.00 | 0.00 | 0.36 | 0.00 | 0.00 | 0.00 | 0.00 | 0.00 | 0.00 |
| *Planctomycetes | 0.18 | 1.27 | 1.09 | 0.00 | 0.91 | 0.73 | 0.64 | 0.00 | 0.18 | 0.36 | 0.00 | 0.46 |
| *Tenericutes | 0.18 | 0.00 | 0.00 | 0.64 | 0.00 | 0.00 | 0.00 | 0.00 | 0.00 | 0.00 | 0.00 | 0.00 |
| **Bacteroidetes** | 0.09 | 4.48 | 3.38 | 4.18 | 1.51 | 3.40 | 0.94 | 0.92 | 3.27 | 1.18 | 0.76 | 0.70 |
| **Fibrobacteres** | 0.05 | 0.00 | 0.86 | 0.12 | 0.15 | 0.60 | 0.00 | 0.00 | 0.00 | 0.14 | 0.00 | 0.00 |
| **Gemmatimonadetes** | 0.05 | 0.00 | 0.00 | 0.24 | 0.08 | 0.00 | 0.05 | 0.08 | 0.00 | 0.00 | 0.00 | 0.00 |
| **Fusobacteria** | 0.04 | 0.34 | 0.12 | 0.12 | 0.00 | 0.23 | 0.00 | 0.13 | 0.36 | 0.18 | 0.04 | 0.00 |
| **Woesearchaeota(DHVEG-6)** | 0.03 | 0.00 | 0.00 | 0.00 | 0.00 | 0.00 | 0.00 | 0.00 | 0.00 | 0.00 | 0.00 | 0.00 |
| **Acidobacteria** | 0.03 | 0.34 | 0.68 | 0.48 | 0.45 | 0.23 | 0.10 | 0.00 | 0.00 | 0.32 | 0.04 | 0.00 |
| **Aminicenantes** | 0.03 | 0.00 | 0.00 | 0.00 | 0.00 | 0.00 | 0.00 | 0.00 | 0.00 | 0.00 | 0.00 | 0.00 |
| **Candidate division SR1** | 0.03 | 0.14 | 0.00 | 0.00 | 0.00 | 0.00 | 0.00 | 0.00 | 0.00 | 0.00 | 0.00 | 0.00 |
| **Chlamydiae** | 0.03 | 0.00 | 0.00 | 0.06 | 0.00 | 0.00 | 0.00 | 0.00 | 0.00 | 0.00 | 0.00 | 0.00 |
| **Deinococcus-Thermus** | 0.03 | 0.34 | 0.00 | 0.12 | 0.08 | 0.00 | 0.00 | 0.00 | 0.00 | 0.00 | 0.02 | 0.02 |
| **Omnitrophica** | 0.03 | 0.00 | 0.00 | 0.00 | 0.15 | 0.00 | 0.00 | 0.00 | 0.00 | 0.00 | 0.00 | 0.00 |
| **Spirochaetae** | 0.03 | 0.28 | 0.43 | 0.12 | 0.30 | 0.51 | 0.10 | 0.21 | 0.36 | 0.07 | 0.07 | 0.07 |
| **Chlorobi** | 0.01 | 0.00 | 0.37 | 0.30 | 0.00 | 0.23 | 0.15 | 0.04 | 0.00 | 0.07 | 0.00 | 0.05 |
| **Cloacimonetes** | 0.01 | 0.14 | 0.00 | 0.00 | 0.00 | 0.00 | 0.00 | 0.00 | 0.00 | 0.00 | 0.00 | 0.00 |
| **Actinobacteria** | 0.00 | 0.00 | 0.00 | 0.48 | 0.38 | 0.55 | 0.10 | 0.29 | 0.36 | 0.07 | 0.02 | 0.02 |
| **Aerophobetes** | 0.00 | 0.00 | 0.00 | 0.00 | 0.00 | 0.00 | 0.00 | 0.00 | 0.18 | 0.00 | 0.00 | 0.00 |
| **Armatimonadetes** | 0.00 | 0.00 | 0.00 | 0.06 | 0.00 | 0.00 | 0.00 | 0.00 | 0.18 | 0.00 | 0.00 | 0.00 |
| **Atribacteria** | 0.00 | 0.00 | 0.00 | 0.00 | 0.00 | 0.00 | 0.10 | 0.00 | 0.00 | 0.00 | 0.00 | 0.00 |
| **Deferribacteres** | 0.00 | 0.00 | 0.06 | 0.00 | 0.00 | 0.00 | 0.00 | 0.00 | 0.00 | 0.00 | 0.00 | 0.00 |
| **Elusimicrobia** | 0.00 | 0.00 | 0.00 | 0.12 | 0.00 | 0.00 | 0.00 | 0.00 | 0.00 | 0.00 | 0.00 | 0.00 |
| **Gracilibacteria** | 0.00 | 0.00 | 0.00 | 0.00 | 0.00 | 0.09 | 0.00 | 0.00 | 0.00 | 0.00 | 0.00 | 0.00 |
| **Hydrogenedentes** | 0.00 | 0.00 | 0.00 | 0.12 | 0.00 | 0.09 | 0.00 | 0.00 | 0.00 | 0.04 | 0.00 | 0.00 |
| **Latescibacteria** | 0.00 | 0.00 | 0.00 | 0.12 | 0.00 | 0.00 | 0.00 | 0.00 | 0.00 | 0.00 | 0.00 | 0.00 |
| **Nitrospirae** | 0.00 | 0.00 | 0.00 | 0.00 | 0.00 | 0.00 | 0.00 | 0.00 | 0.00 | 0.04 | 0.00 | 0.00 |
| **Synergistetes** | 0.00 | 0.00 | 0.00 | 0.00 | 0.00 | 0.00 | 0.00 | 0.00 | 0.00 | 0.00 | 0.00 | 0.05 |
| **TM6** | 0.00 | 0.00 | 0.12 | 0.00 | 0.00 | 0.05 | 0.00 | 0.00 | 0.00 | 0.00 | 0.00 | 0.00 |
| *Deferribacteres | 0.09 | 0.00 | 0.09 | 0.00 | 0.00 | 0.00 | 0.00 | 0.00 | 0.00 | 0.00 | 0.00 | 0.00 |
| *Fibrobacteres | 0.09 | 0.00 | 1.00 | 0.00 | 0.18 | 0.73 | 0.00 | 0.00 | 0.00 | 0.00 | 0.00 | 0.00 |
| *Fusobacteria | 0.09 | 0.36 | 0.09 | 0.18 | 0.00 | 0.27 | 0.00 | 0.27 | 0.36 | 0.27 | 0.00 | 0.00 |
| *Spirochaetae | 0.09 | 0.09 | 0.64 | 0.09 | 0.27 | 0.64 | 0.09 | 0.18 | 0.36 | 0.00 | 0.00 | 0.09 |
| *Acidobacteria | 0.00 | 0.27 | 0.45 | 0.45 | 0.45 | 0.36 | 0.00 | 0.00 | 0.00 | 0.00 | 0.00 | 0.00 |
| *Actinobacteria | 0.00 | 0.00 | 0.00 | 0.18 | 0.45 | 0.55 | 0.18 | 0.36 | 0.36 | 0.09 | 0.00 | 0.09 |
| *Aerophobetes | 0.00 | 0.00 | 0.00 | 0.00 | 0.00 | 0.00 | 0.00 | 0.00 | 0.18 | 0.00 | 0.00 | 0.00 |
| *Armatimonadetes | 0.00 | 0.00 | 0.00 | 0.09 | 0.00 | 0.00 | 0.00 | 0.00 | 0.18 | 0.00 | 0.00 | 0.00 |
| *Atribacteria | 0.00 | 0.00 | 0.00 | 0.00 | 0.00 | 0.00 | 0.18 | 0.00 | 0.00 | 0.00 | 0.00 | 0.00 |
| *Bacteroidetes | 0.00 | 4.64 | 3.45 | 3.82 | 1.64 | 2.73 | 1.09 | 1.19 | 3.27 | 0.73 | 0.27 | 1.18 |
| *Candidate division SR1 | 0.00 | 0.18 | 0.00 | 0.00 | 0.00 | 0.00 | 0.00 | 0.00 | 0.00 | 0.00 | 0.00 | 0.00 |
| *Chlamydiae | 0.00 | 0.00 | 0.00 | 0.09 | 0.00 | 0.00 | 0.00 | 0.00 | 0.00 | 0.00 | 0.00 | 0.00 |
| *Chlorobi | 0.00 | 0.00 | 0.36 | 0.36 | 0.00 | 0.18 | 0.18 | 0.00 | 0.00 | 0.09 | 0.00 | 0.00 |
| *Chloroflexi | 0.00 | 0.64 | 1.45 | 4.91 | 0.36 | 0.18 | 0.46 | 1.37 | 0.18 | 1.45 | 0.00 | 0.36 |
| *Cloacimonetes | 0.00 | 0.18 | 0.00 | 0.00 | 0.00 | 0.00 | 0.00 | 0.00 | 0.00 | 0.00 | 0.00 | 0.00 |
| *Deinococcus-Thermus | 0.00 | 0.36 | 0.00 | 0.00 | 0.09 | 0.00 | 0.00 | 0.00 | 0.00 | 0.00 | 0.00 | 0.09 |
| *Elusimicrobia | 0.00 | 0.00 | 0.00 | 0.18 | 0.00 | 0.00 | 0.00 | 0.00 | 0.00 | 0.00 | 0.00 | 0.00 |
| *Firmicutes | 0.00 | 1.55 | 0.18 | 1.18 | 0.00 | 2.00 | 0.09 | 1.28 | 0.00 | 0.27 | 0.00 | 0.00 |
| *Gemmatimonadetes | 0.00 | 0.00 | 0.00 | 0.27 | 0.09 | 0.00 | 0.09 | 0.09 | 0.00 | 0.00 | 0.00 | 0.00 |
| *Gracilibacteria | 0.00 | 0.00 | 0.00 | 0.00 | 0.00 | 0.09 | 0.00 | 0.00 | 0.00 | 0.00 | 0.00 | 0.00 |
| *Hydrogenedentes | 0.00 | 0.00 | 0.00 | 0.09 | 0.00 | 0.09 | 0.00 | 0.00 | 0.00 | 0.00 | 0.00 | 0.00 |
| *Latescibacteria | 0.00 | 0.00 | 0.00 | 0.09 | 0.00 | 0.00 | 0.00 | 0.00 | 0.00 | 0.00 | 0.00 | 0.00 |
| *Nitrospirae | 0.00 | 0.00 | 0.00 | 0.00 | 0.00 | 0.00 | 0.00 | 0.00 | 0.00 | 0.09 | 0.00 | 0.00 |
| *Omnitrophica | 0.00 | 0.00 | 0.00 | 0.00 | 0.18 | 0.00 | 0.00 | 0.00 | 0.00 | 0.00 | 0.00 | 0.00 |
| *Synergistetes | 0.00 | 0.00 | 0.00 | 0.00 | 0.00 | 0.00 | 0.00 | 0.00 | 0.00 | 0.00 | 0.00 | 0.09 |
| *TM6 | 0.00 | 0.00 | 0.09 | 0.00 | 0.00 | 0.09 | 0.00 | 0.00 | 0.00 | 0.00 | 0.00 | 0.00 |
